# Leaving the Homestead: Examining the Role of Relative Deprivation, Social Trust, and Urban Integration among Rural Farmers in China

**DOI:** 10.3390/ijerph191912658

**Published:** 2022-10-03

**Authors:** Wentao Si, Chen Jiang, Lin Meng

**Affiliations:** School of Public Administration, Shandong Normal University, Jinan 250014, China

**Keywords:** relative deprivation, social trust, urban integration, homestead, willingness to withdraw, Jinan

## Abstract

Actively promoting the orderly and voluntary withdrawal of idle rural house bases and effectively activating “sleeping” land assets are considered important measures to accelerate the modernization of agriculture and rural areas, as well as promoting the integrated development of urban and rural areas. However, few studies have focused on the superimposed effects of negative psychological and social environmental factors on the willingness of farmers to withdraw from their homestead. Therefore, we conducted this study in order to clarify the logical ideas of rural home base withdrawal, analyze the factors that influence the decision of home base withdrawal behavior of interest subjects, and provide a scientific decision basis for promoting rural home base withdrawal and accelerating the process of transferring citizens of agricultural population in terms of policies, measures, and paths, so as to design and develop an incentive mechanism for home base withdrawal of citizens of agricultural transfer population. The results of the study show that: (1) the findings indicate that social deprivation, economic deprivation, and emotional deprivation all significantly and negatively affect farmers’ willingness to withdraw from their homesteads; (2) social trust plays a mediating role between relative deprivation and farmer homestead withdrawal behavior; and (3) urban integration plays a moderating role between social and emotional deprivation and the social trust of farmers, but does not have a significant moderating role in the relationship between economic deprivation and social trust. Furthermore, it plays a moderating role between all deprivation factors (i.e., economic, social, and emotional deprivation) and farmer homestead withdrawal behavior. This study aims to provide useful guidance and policy suggestions for optimizing policies related to farmer homestead withdrawal behaviors, and for scientifically designing the homestead withdrawal mechanism and policy system.

## 1. Introduction

The root cause of the lagging process regarding the urban citizenship of the agricultural migrant population is considered to be the “umbilical cord” relationship between farmers and rural residential bases [1]. With the continuous promotion of new types of urbanization, a large number of migrant agricultural workers are expected to flow into cities, with a total of 292.51 million migrant workers in China by 2021 [2]. According to statistics, by the end of 2021, the urbanization rate of China’s resident population had reached 64.72%, but the urban household registration rate was only 46.7% [3]. The current registered residence system in China divides the household registration into urban household registration and rural household registration according to the relationship of blood inheritance and geographical location. This urban–rural “amphibious” living pattern of rural laborers has created a dilemma, in which the rigid demand for urban land co-exists with the wasteful use of rural land [4]. According to data from the Ministry of Agriculture and Rural Development, the rural labor force has been living in rural areas for more than 20 years. According to the data of the Ministry of Agriculture and Rural Affairs, the area of rural house bases is 168 million mu, accounting for 48.62% of the collective construction land area. According to the relevant data, there are 261 million migrant workers and their accompanying family members who have become permanent residents in urban areas, and the idle rate of rural residential bases is as high as 17.6% [5]. This large number of unused rural residential bases not only leads to a waste of land resources and various safety hazards, but also seriously affects the improvement of the rural living environment and the promotion of rural urbanization [6]. In order to resolve the dilemma of idle and inefficient use of residential bases, in 2004, the State Council already pointed out in the Central Document No. 28 that it advocates the moderate adjustment of rural construction land, and the moderate increase of urban construction land should be linked with the moderate reduction of rural construction land. In 2008, the State Council clearly put forward the policy of “urban-rural linkage increase/decrease” in the “Management Measures of Urban-Rural Construction Land Increase/Decrease Pilot”. In 2016, the State Council issued “Several Opinions on Deepening the Construction of a New Type of Urbanization” (No.8 Document of State Council [2016]), which pointed out that “exploring the mechanism of voluntary and compensated withdrawal of farmers’ rights to land contract, use homestead and collective income distribution”. In 2022, the No. 1 document of “the Central Committee of the Communist Party of China (CPC) and the State Council on the key work of comprehensive promotion of rural revitalization in 2022” further emphasized “prudently promote the pilot reform of the rural homestead system, and standardize the registration of housing land as one of the homestead rights”. It points out the direction for regulating rural construction land, promoting the voluntary and compensated withdrawal of rural land rights and interests of the agricultural transfer population, and implementing the withdrawal mechanism of rural homestead in the context of citizenship. The newly amended Land Management Law in 2019 stipulates that the state will allow rural villagers who have settled in cities to withdraw from their residential bases voluntarily and be compensated, according to the law. However, the results of a large number of surveys regarding the willingness to withdraw from residential bases have shown that the percentage of farmers who settle in cities and are unwilling to withdraw from their residential bases is 50–70% [7]. As the voluntary principle is the basis for the implementation of the homestead withdrawal system [8], it is of great practical significance to study the influencing mechanisms of the decision to withdraw from residential bases by farmers who have settled in cities in depth, in order to scientifically design the mechanism for voluntary and paid withdrawal from residential bases, thus encouraging farmers who have settled in cities to withdraw from their residential bases voluntarily and steadily promoting the reform of the rural residential base system.

In the existing relevant literature, scholars have mainly focused on value perception [9], risk expectation [10], ownership [11], local attachment [12], and so on, and have explored the various influences on the willingness of urban farmers to withdraw from their residential bases. The decision to voluntarily withdraw from residential bases is based on a rational analysis of the trade-off between pros and cons; however, as there is no uniform standard for the judgment of pros and cons, this judgment may be highly subjective [13]. The decision of farmers to voluntarily withdraw from their residential land is based on rational analysis, instinctive psychological factors (e.g., internal emotions, desires, and perceptions), and their external social environment [14]. Therefore, in this study, we analyze the interactions between and influence of emotional, psychological, and social environment factors on the willingness of farmers in cities to withdraw from their homestead bases.

From the perspective of the armer’s own internal psychological factors, 55.98% of farmers subjectively believe that homestead bases are privately owned by the farmers [4]. However, from the perspective of legal system, the ownership of rural homestead in China belongs to the collective, and farmers only have the right to use the homestead. This is because rural homestead assume a variety of functions, such as production, living, and emotional belonging, to which farmers assign higher value expectations [15]. Withdrawal from the rural homestead is a major problem, which entails the loss of these functions and rights. Therefore, farmers will repeatedly make psychological trade-offs when withdrawing from their homesteads, which makes farmers have a strong sense of relative deprivation which, in turn, inhibits their homestead withdrawal behavior [16]. The relative dispossession of farmers arises from their sense of dispossession. The sense of relative deprivation in farmers originates from a loss of functional dependence on the homestead, which also reflects their concerns regarding access to material resources, social relations, and development opportunities after losing the functional rights and interests carried by the homestead under the conditions of relatively institutionalized social inequality [17].

An individual’s life situation is no longer confined to the private world but is increasingly influenced by the broader social context and social structure. In other words, the relative sense of deprivation produced by social comparison of individuals is not only related to their own conditions, but also, to some extent, is the result of the social environment. In terms of the factors that influence the willingness of farmers to withdraw from their homestead, the following two aspects are the most important.

First, the behavior of farmers will be influenced by external social environment factors (i.e., social trust). With the implementation of the rural revitalization strategy and the construction of rural civilization, the social trust among farmers has been enhanced. Social trust relationships reflect an individual’s rights and obligations to others or social organizations to honor the relationship. So, does the level of social trust affect the homestead withdrawal behavior of farmers? Does the level of social trust play a mediating role in the relative sense of deprivation on their willingness to withdraw from their homestead? Does the sense of relative deprivation lead to a decrease in trust in the government, which in turn has an impact on the willingness of rural residents to withdraw from their homestead? These questions are worth exploring in depth. Second, homestead withdrawal requires farmers to complete urban integration into a new place. Urban integration does not only involve economic integration, in terms of labor and employment, economic income, and living conditions but, more importantly, it involves a higher level of integration in social and psychological aspects. This is mainly reflected in the conceptual acceptance and recognition of urban culture, such as the customs, behavioral norms, and values in cities. This cultural and psychological urban integration status reflects the degree of attachment and psychological distance of urban farmers to the city and influences their behavior and decision making. Urban integration can lead to a positive interaction between individual development and social development. Therefore, in this paper, we incorporate urban integration into the considered analytical framework and explore the moderating effect of urban integration on the homestead withdrawal behavior of farmers.

Based on this, we conducted this study in order to clarify the logical ideas of rural home base withdrawal, analyze the factors that influence the decision of home base withdrawal behavior of interest subjects, and provide a scientific decision basis for promoting rural home base withdrawal and accelerating the process of transferring citizens of agricultural population in terms of policies, measures and paths, so as to design and develop an incentive mechanism for home base withdrawal of citizens of agricultural transfer population. We selected some urban migrants for empirical research. The urban migrant group studied in this paper refers to those who move from rural to urban areas for a long period of time, referred to as “rural migrants”. This concept breaks through the limitations of the concept of “migrant workers” used in earlier studies, based on the background of the urban–rural dual structure, and is consistent with the trend characteristics of the rural population moving to the city to work and live, in the context of accelerating the citizenship of the migrant agricultural population in the new urbanization construction stage. The concept of “migrant workers” has been widely used in academic circles, as it is in line with the trend of long-term, family-oriented, and sedentary living of the rural population in the context of accelerating the citizenship of the agricultural transfer population in the new urbanization stage. Our empirical study investigates the intrinsic mechanisms of relative deprivation that influence the withdrawal of rural households from their homestead and identifies the mediating effect of social trust and the moderating effect of urban integration.

## 2. Literature Review and Research Hypothesis

### 2.1. Connotation Definition

#### 2.1.1. Connotation of Relative Deprivation

The sense of relative deprivation is a subjective perception that individuals or groups find themselves in a disadvantaged position through comparison, which includes not only cognitive components of social comparison, but also emotional components such as anger and dissatisfaction, and even behavioral components such as complaints, conflicts, and revenge. Disadvantaged individuals may experience a sense of deprivation of basic rights during the process of social comparison, and this sense of deprivation easily leads to negative effects on their psychological and behavioral adaptation. Relative deprivation can lead to a variety of negative problem behaviors [18]. As a negative subjective experience, the sense of relative deprivation—as an evaluation of one’s deprived rights and low social status—can easily produce frustration and depression, thus increasing the risk of externalizing problem behaviors [19]. Based on the multiple functions of homestead, the relative deprivation of farmers can be divided into economic deprivation, social deprivation, and emotional deprivation [20,21,22]. Previous studies have taken the hypothesis of rational economic man of farmers as the premise of the study and so could not comprehensively understand the homestead withdrawal decision behaviors of farmer, and cannot explain the fact why many farmers are still reluctant to withdraw from their homestead, even after their human capital situation (e.g., education and employment) has been improved. Recent studies in sociology, psychology, or consumer behavior fields have found that, when individuals or groups experience a sense of relative deprivation, obtaining corresponding psychological compensation is their fundamental core demand [23]. This is also the solution to promoting the voluntary withdrawal from residential bases of urban farmers.

#### 2.1.2. The Connotation of Homestead to Farmers

Residential land is the land used by farmers to build houses, buildings, and facilities related to residential life [24]. The main characteristics of the Chinese residential land system can be described as: “collective ownership, use by members, free of land price, and long-term occupation” [25]. Although the current law stipulates that residential bases are collectively owned, in practice they have been occupied and used by different farmers separately, and the concept of ownership of residential bases by farmers has long been rather vague, or the concept of thinking that they own them has not fundamentally changed [26]. A homestead is land for living acquired by farmers, as members of collective economic organizations without compensation, thus having the nature of welfare distribution. When they own a residential base, farmers not only live there and store crops and farming tools, but also generate other productive collateral benefits, such as the convenience of growing fruits and vegetables on the residential base, handicraft workshop production, and so on [27]. At the same time, the sense of security brought by having a place to live and the psychological satisfaction of building a relationship with the surrounding environment are also important components of family welfare for farmers, which are shared by family members [28]. From this perspective, the relationship between farmers and their homestead is not only economic, but also embedded with socio-cultural and psycho-emotional factors, which determines the complexity of homestead withdrawal decisions. Therefore, when farmers choose to withdraw from their homestead, it means that these benefits attached to the homestead will also be lost [29]. Thus, the homestead is currently an important place for a significant majority of farmers to live and survive, and the choice to withdraw from the homestead or not will affect the overall welfare of their households.

#### 2.1.3. The Connotation of Urban Integration

The urban integration of migrant farmers refers to the process by which farmers establish their economic status in the city, adapt to urban social norms and security, and finally achieve urban psychological identity [30,31]. In this study, we analyze the urban integration of migrant farmers in three dimensions: Economic integration, social integration, and psychological integration. Economic integration refers to the integration of farmers into the economic conditions of urban life when they move from rural to urban areas, which is the basis of urban integration of migrant farmers [30,32]. Social integration refers to the establishment of positive social networks with people around them (e.g., colleagues, leaders, neighbors) and the creation of continuous interactive behaviors [33]. Psychological integration emphasizes the acceptance and recognition of the city by the farmers; that is, the farmers have a sense of belonging to the city, are more willing to live in urban areas, and have a sense of citizenship in the city. The completion of psychological integration is the completion of the process of transforming farmers from rural to urban people, thus realizing urban integration in the true sense [34].

#### 2.1.4. The Connotation of Social Trust

Social trust is formed between members of society based on a combination of morality and interest and can be viewed as a psychological contract of mutual expectation, mutual trust, and interdependence. Social trust, as a systemic trust, is also an important influential factor affecting the social mindset of farmers [35]. Social trust is an implicit commitment among, is practiced by, and its benefits act on all members of society, thus having the properties of public goods. When every member of the society remembers the promise of “all for one, one for all” and imparts general trust to the society, the cost of interaction is reduced and the efficiency of interaction is improved, helping to build a stable cooperation rule and reciprocity mechanism, thus improving the efficiency of cooperation [36]. From a sociological perspective, this study views social trust as a social phenomenon based on jurisprudence (regulatory system) or ethics (social and cultural norms). On this basis, two different types of social trust are distinguished: juridical social trust and ethical social trust. The former originates from the regulatory system [37], while the latter is based on moral norms and values [38]. Specifically, the social trust of urban farmers also includes the following two levels: legal social trust (i.e., trust in government and law) and moral social trust (i.e., trust in the urban environment and social justice) [37,38,39,40].

### 2.2. Theoretical Assumptions and Analysis

#### 2.2.1. The Direct Effect of Relative Deprivation on Farmer Homestead Withdrawal Behavior

Although the institutional arrangements for the collectivization of homestead ownership are becoming clearer at the legal system level, the long-term factual possession and “monopoly” use of homesteads by farmers have not changed their “quasi-ownership” of homesteads, due to policy changes [41]. This causes farmers to assign higher value expectations to these functions, and withdrawal from the homestead means the loss of these functional rights and interests. Longitudinal comparison before and after the withdrawal causes farmers to have a strong sense of relative deprivation [14] which, in turn, generates negative perceptions and discourages farmers from withdrawing from their homesteads.

Based on the above analysis, the following hypotheses are proposed.

**H1.**  *Relative deprivation has a negative effect on the willingness of farmers to withdraw from their homesteads.*

**H1a.**  *The sense of economic deprivation has a negative effect on the willingness of farmers to withdraw from their homesteads.*

**H1b.**  *Social deprivation has a negative effect on the willingness of farmers to withdraw from their homesteads.*

**H1c.**  *Affective deprivation has a negative effect on the willingness of farmers to withdraw from their homesteads.*

#### 2.2.2. The Mediating Effect of Social Trust

(1) The direct effect of relative deprivation on social trust.

From the perspective of population mobility, the sense of relative deprivation acts as a disincentive to labor mobility. For incoming urban farmers, the sense of relative deprivation reduces their social trust and increases the social cost of citizenship, to a certain extent [42]. Farmers are rational, mostly risk-averse, and conservative, such that a higher sense of relative deprivation reduces their expectations of future employment, income, and a better life, which adversely affects their willingness to withdraw from the homestead [43]. Related to this, the satisfaction of farmers with their socio-economic status affects their evaluation of the performance, management, and governance ability of government departments which, in turn, affects their level of social trust; that is, an individual’s sense of individual deprivation, and the resulting negative emotional reactions and adverse social perceptions, may trigger their distrust of the legitimacy of institutional norms [44]. From the above, we can hypothesize that:

**H2.**  *The higher the relative deprivation of farm households, the lower the level of their social trust.*

**H2a.**  *The higher the sense of economic deprivation of farmers, the lower their level of social trust.*

**H2b.**  *The higher the sense of social deprivation of farmers, the lower their level of social trust.*

**H2c.**  *The higher the emotional deprivation of farmers, the lower their level of social trust.*

(2) The direct influence of social trust on farmer homestead withdrawal behavior.

The higher the degree of social trust of farm households, the lower their expectation of opportunistic behavior of others, and the lower the expectation of risk of loss they face. The specific impact paths are described as follows: (1) An increase in social trust can enhance the trust of farmers in the government’s commitment, thus reducing their worries about various risks after homestead withdrawal [45]. (2) In the implementation of homestead withdrawal policies, farmers often do not have a direct dialogue with the government and, instead, are represented by village cadres or township cadres to carry out the policy. (3) After moving into the city, if the social trust of the farmers is high, they will believe that the relevant government departments can give equal civil and social rights to the migrant population, eliminate institutional exclusion, and give priority to solving their work injury insurance and medical protection for major diseases, as well as gradually solving the problem of pension protection [46].

**H3.**  *Social trust has a positive effect on the willingness of farmers to withdraw from their homesteads.*

(3) The mediating effect of relative deprivation between the effect of social trust and farmer willingness to withdraw from their homestead.

The decrease in trust in society triggered by the sense of relative deprivation, in turn, has an impact on the willingness of farmers to withdraw from their homestead. The functions of local governments are closely related to the productive and living activities of rural residents, and directly affect the rational distribution of social resources, social equity, and justice [47]. The sense of relative deprivation means that rural households have not been provided with social resources within a reasonable expectation within the institutionalized environment [48], which will lead to the farmers doubting the credibility of the government, in turn affecting the willingness of farmers to withdraw from their homestead.

Accordingly, the following research hypotheses were formed.

**H4.**  *Social trust mediates the relationship between relative deprivation perception and the willingness of farmers to withdraw from their homestead.*

**H4a.**  *Social trust mediates the relationship between feelings of economic deprivation and the willingness of farmers to withdraw from their homestead.*

**H4b.**  *Social trust mediates the relationship between feelings of social deprivation and the willingness of farmers to withdraw from their homestead.*

**H4c.**  *Social trust mediates the relationship between feelings of emotional deprivation and the willingness of farmers to withdraw from their homestead.*

#### 2.2.3. Moderating Effects of Urban Integration

(1) The moderating role of urban integration in the relationship between the sense of relative deprivation and willingness to withdraw from their homestead.

According to neoclassical economic theory, farmers, as “rational economic agents”, are willing to give up their homesteads only when the risk is within the tolerable range and the total benefit of land withdrawal is significantly higher than the total cost [49]. The purpose of withdrawal is to maximize economic and social returns. High-quality urban integration can promote equal opportunities in employment, housing, medical care, and education, as well as reducing the loneliness associated with discrimination and exclusion from citizenship and rights [50]. Thus, it weakens the negative impact of relative deprivation of farmers, in economic, social, and emotional terms, on their homestead withdrawal behavior, thus compensating for the relative deprivation in the sense of loss of functional rights and interests regarding the homestead. Thus, it can be hypothesized that urban integration plays an important moderating role in the effect of relative deprivation on farmer homestead withdrawal behavior.

Therefore, the following hypotheses are proposed.

**H5.**  *Urban integration has a positive moderating effect on the relative sense of deprivation affecting the homestead withdrawal behavior of farmers.*

**H5a.**  *Urban integration has a positive moderating effect on the sense of economic deprivation affecting the homestead withdrawal behavior of farmers.*

**H5b.**  *Urban integration has a positive moderating effect on the sense of social deprivation affecting the homestead withdrawal behavior of farmers.*

**H5c.**  *Urban integration has a positive moderating effect on the sense of emotional deprivation affecting the homestead withdrawal behavior of farmers.*

(2) The moderating role of urban integration in the relationship between relative deprivation and social trust.

When farmers become urban workers and perform the same work as urban workers, they still do not enjoy the same social security benefits as urban workers, thus creating multiple compensation systems. China’s current social security system, such as pension insurance, unemployment insurance and medical insurance, requires a local registered residence, which obviously only protects the urban population and excludes the vast rural population. Migrant workers in the cities cannot enter the relatively complete urban social security system, they do not have minimum subsistence money and unemployment protection, and if they cannot work or do not have a job, they can only starve or go back to the countryside [51]. In the comparison with urban workers, the group of rural migrants moving to urban areas have a sense of “relative deprivation”, which not only makes them feel distrustful and lack a sense of identity with urban areas, but also restricts the flow of labor and affects the effective allocation of labor resources.

The social trust relationship involves the cashing in of the rights and obligations of the individual urban migrant to others or social organizations [52]. Social trust is based on the norm of mutual benefit and reciprocity, which means that interest relationships are important factors affecting the operation of trust [53]. Such interest relationships include economic, social and psychological aspects. The interactions between farmers and urbanites, their feelings during such interactions, and the information they obtain about the society provide an important basis for building social trust [54]. Therefore, the following hypothesis was proposed:

**H6.**  *Urban integration has a positive moderating effect on the relationship between relative deprivation and social trust.*

**Figure 1:**
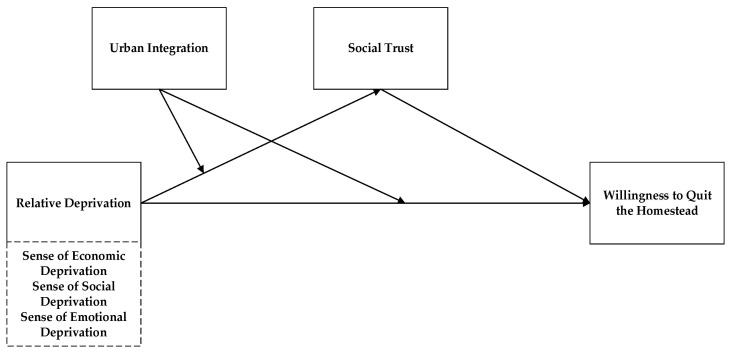
Theoretical framework of the study.

In summary, in this paper, we attempt to incorporate the sense of relative deprivation, social trust, urban integration, and homestead withdrawal willingness into the same analytical framework (Figure 1), thus constructing a theoretical model of farmer homestead withdrawal willingness in order to provide a new perspective for the study of farmer homestead withdrawal in the context of homestead system reform. The model better utilizes the explanatory framework of psychological factors of farmers to analyze how the willingness of farmers to withdraw from their homesteads is generated. The model consists of three parts: The first part considers the role of relative deprivation on the willingness of farmers to withdraw from their homesteads; the second part considers the mediating role of social trust between relative deprivation and the willingness to withdraw; and the third part considers the moderating role of urban integration between relative deprivation and willingness to withdraw, also considering the moderating role of urban integration in the relationship between relative deprivation and social trust (see Figure 1).

## 3. Study Design

### 3.1. Research Methodology

The Amos 21.0 (IBM, New York, USA) software was applied to analyze the effect of each dimension of relative deprivation on the willingness to withdraw behavior. Each latent variable was measured through multiple entries in the scale. To further validate the mediating effect of perceived benefits, the Bootstrap moderated mediation test method approach was used to validate the mediating effect of social trust, while the moderating effect of urban inclusion was tested using multi-variate cascade regression.

### 3.2. Variable Selection

#### 3.2.1. Independent Variable: Sense of Relative Deprivation

With reference to Smith [55], Osborne [56], and Xiong M. [57], we designed a measure of farmer relative deprivation including nine indicators. Among them, economic deprivation indicators included changes in perceptions of agricultural production, new house acquisition, and production costs before and after retirement; social deprivation indicators included changes in perceptions of employment, pension, and urban treatment before and after retirement; and emotional deprivation indicators included changes in perceptions of belonging, loneliness, and local attachment before and after retirement. The indicators were all on a 5-point Likert scale, with 1 indicating “strongly disagree” and 5 indicating “strongly agree”. The higher the score, the greater the relative deprivation of the farmers (Table 1).

#### 3.2.2. Mediating Variable: Social Trust

Specifically for migrant workers, social trust includes at least two levels: legal social trust (i.e., trust in government and law) [58,59,60] and moral social trust (i.e., trust in the urban environment and social justice) [61]. The four dimensions of trust were measured by the eight questions described in Table 2. These indicators were also on a 5-point Likert scale, with 1 indicating “strongly disagree” and 5 indicating “strongly agree”. Again, the higher the score, the greater the relative deprivation of the farmers (Table 2).

#### 3.2.3. Moderating Variable: Urban Integration

With reference to Li, H.B. et al. [62], Tian, M. [63], and Zhao, Q. [64], the indicators to measure the urban integration of migrant workers were selected from three dimensions: Economic integration, social integration, and psychological integration. A total of nine measurement questions were used (see Table 3), which were measured on a 5-point Likert scale, with 1 indicating “strongly disagree” and 5 indicating “strongly agree”. The higher the score, the greater the degree of urban integration of rural households (Table 3).

#### 3.2.4. Dependent Variable: Willingness to Withdraw from the Homestead

Willingness to withdraw from the homestead is a psychological state. With reference to Kwong Fo-yeon [65], Huang Yanxin [66], Peng Changsheng [67], and other scholars, the following three questions were chosen to measure willingness to withdraw: “I am willing to exchange my homestead for a government-unified resettlement house”, “I am willing to exchange my homestead for a commercial house of the same area”, and “I am willing to exchange my homestead for financial compensation”. The indicators were measured on a 5-point Likert scale, with 1 indicating “strongly disagree” and 5 indicating “strongly agree”. The higher the score, the stronger the willingness to withdraw from the residential base (Table 4).

### 3.3. Study Area and Data Acquisition

The data used in this paper came from a sample survey of farmers moving into the city of Jinan, conducted by the subject group. Jinan, one of the 15 sub-provincial cities in China, has been one of the major inflow areas for migrant population in the country. In April 2020, Jinan introduced the “Measures to Deepen the Reform of Household Registration System and Accelerate the Gathering of Talents”, which completely abolished the restrictions on settling in urban areas and townships for foreigners who are willing to work and live in Jinan. Thus, Jinan became the third provincial capital in China, after Shenyang and Nanchang, to have “zero threshold” for settling down. The urbanization rate of the resident population in Jinan reached 74.2%, 10.48 percentage points higher than the national average. The existing urbanized farmers in Jinan are mainly distributed in six major industries: manufacturing; transportation, warehousing, and postal services; construction; accommodation and catering; wholesale and retail; and residential services and repair [68]. Therefore, the findings from the case study of Jinan City are considered to be representative of the urban integration and willingness to withdraw from residential bases in large- and medium-sized cities across China, in terms of the number and employment patterns of urban migrant farmers.

The data for the empirical study in this paper were obtained from a survey questionnaire. In order to collect data, we formulated the “Study on the Willingness of Farmers in Jinan to Withdraw from Homesteads” which involved a total of 37 survey items and set up a survey team of 30 students. For some migrant workers who could not understand the content of the questionnaire correctly, we used the method of dictating the questionnaire questions to the respondents and recording the answers. In addition, in order to reduce the influence of social expectation bias, we explained to respondents in the introduction section of the questionnaire that this survey is anonymous and that the data will only be used for academic research. We used a combination of stratified sampling and random sampling to distribute and research the questionnaire. The principles of quotas were as follows: Firstly, districts were used as primary sampling units in Jinan city, and five districts—namely, Lixia district, Shizhong district, Licheng district, Huaiyin district, and Changqing district—were selected as sample survey areas, according to the regional economic development indicators. Among them, Lixia district is the district with the highest level of economic development in Jinan city; Shizhong district, Licheng district, and Huaiyin district are in the middle level; and Changqing district presents relatively weak economic development. Secondly, we selected 240–360 farmers in each district who moved to the city (120–180 in commercial complexes, 72–108 in construction projects, and 48–72 in factories). Next, two commercial complexes, two construction projects, and two factories were randomly selected in each district and we conducted the survey in the form of one-on-one questionnaire interviews. From the perspective of the industries engaged in by migrant workers, the number of people engaged in the secondary industry accounts for about 50.56%, of which the manufacturing industry accounts for about 19.45% and the construction industry accounts for about 31.11%. The tertiary industry accounts for about 49.44%, and these migrant workers mainly work in six major industries, namely manufacturing, transportation, warehousing and postal services, construction, accommodation and catering, wholesale and retail, and resident service repair.

The urban farmers studied were those who had a rural household registration and a rural residential base, but who had left their hometown to work in a non-agricultural industry in Jinan, including domestic workers, maintenance workers, restaurant chefs, hotel waiters, laborers, construction workers, store salesmen, and take-away courier workers. In addition, in order to reduce the influence of social expectation bias, the introduction of the questionnaire explained to the respondents that this was an anonymous survey and that the data would only be used for academic research. The data collection was divided into two stages: A pre-survey and a formal survey. A total of 100 questionnaires were returned in the pre-survey stage from May to June 2021, and some items were improved after reliability and validity analyses (Table 5).

## 4. Empirical Tests and Analysis of Results

### 4.1. Structural Equation Model Testing

#### 4.1.1. Reliability and Validity Tests

The Cronbach’s alpha values for sense of economic deprivation, social deprivation, emotional deprivation, social trust, urban integration, and willingness to withdraw were 0.850, 0.897, 0.905, 0.930, 0.954, and 0.835, respectively, all of which were greater than 0.7. It is evident that the questionnaire scale used in this study had good reliability. In addition to this, the CITC (corrected item total correlation) between the observed variables and their latent variables met the requirement of being greater than 0.5, indicating that the questionnaire reliability was good for each latent variable, with a good set of question items.

In order to determine the structural validity of the measurement scale for each latent variable, we used the SPSS 21 software to conduct exploratory factor analysis for each dimensional composition. The initial test results showed that the KMO test value for the survey data was 0.943, which was greater than 0.70, indicating that the questionnaire was suitable for factor analysis. The Bartlett’s sphericity test showed that the approximate chi-square value was 28,856.003, with a significance level of 0.000 (*p* < 0.01). Therefore, the scale was suitable for factor analysis and, therefore, had a good validity.

In the results of the validity test for the overall questionnaire, six factors with eigenvalues greater than one were extracted in the factor analysis, including a factor with an eigenvalue of 5.915, explaining 20.291% of the variance; an “emotional deprivation” factor with an eigenvalue of 1.758, explaining 8.681% of the variance; and a “social deprivation” factor with an eigenvalue of 1.454, explaining 7.778% of the variance. The cumulative explained variance of these six factors was 74.547%, which was greater than the general standard of 60%, such that the validity of the scale was considered good. The loadings of each measure were higher than 0.5, and there were no cases of high loadings for both factors. The measurement items under each dimension were aggregated together, according to the theoretical distribution, indicating that the questionnaire also had good content validity.

To judge whether the validated factor analysis model was valid, we considered various fitting indicators. In particular, the X^2^/df value was 3.216, which is greater than 3; and the RMSEA was 0.041, less than the standard level of 0.08, indicating a good fit. Furthermore, gfi = 0.943, agfi = 0.932, nfi = 0.960, ifi = 0.972, cfi = 0.972, and TLI = 0.969, such that all goodness-of-fit indicators met the general criteria, indicating that the validated factor analysis model developed in this study was valid and matched well with the recovered data (Table 6).

As can be seen from the above table, the standardized factor loadings of each question item ranged from 0.71 to 0.913, which were all greater than 0.5, and the standard error values of SE were all less than the standard value of 0.5, indicating that each question item could explain its dimension well, which echoed the results of exploratory factor analysis and further proved that the validity of the questionnaire was good. The combined reliability CR was greater than 0.7, indicating that all the items in each latent variable could consistently explain the latent variable. The AVE values were all above the standard value of 0.5, indicating that the scale used in this paper had good convergent validity.

#### 4.1.2. Structural Equation Model Fitting Index

The validity of the structural equation model is mainly measured in terms of various fitting indicators. Here, the X^2^/df value was 1.368, which was less than 3; and the RMSEA was 0.034, less than the standard level of 0.08, indicating a good fit. Furthermore, gfi = 0.917, agfi = 0.9, nfi = 0.919, ifi = 0.977, cfi = 0.977, and TLI = 0.974, such that all goodness-of-fit indicators met the general criteria, indicating that the structural equation model developed in this study was valid and fit the recovered data well.

### 4.2. Analysis of Data Results

The AMOS 21.0 (IBM, New York, NY, USA) software was used to conduct the structural equation model path analysis, from which we obtained the structural equation model path coefficient values and CR values shown in Table 7. The path coefficients reflect the relationships and degree of influence between variables, while the critical ratio (CR) can determine whether the regression coefficients are significant or not; generally speaking, the CR value should be greater than or equal to 1.96, which indicates a significant difference at the 0.05 significance level (Table 7).

#### 4.2.1. Path Analysis of the Influence of Relative Deprivation Feeling on the Willingness to Withdraw

The standardized path coefficient of economic deprivation on withdrawal intention was −0.226 (t-value = −5.92; *p* = 0.000 < 0.001), indicating that economic deprivation had a significant negative effect on withdrawal intention. Therefore, 2a is valid. The standardized path coefficient of social deprivation on withdrawal intention was −0.309 (t-value = −8.539; *p* = 0.000 < 0.001), indicating that social deprivation had a significant negative influence on the willingness to withdraw. Thus, H2b is valid. The standardized path coefficient of emotional deprivation on the willingness to withdraw was −0.198 (t-value = −6.557; *p* = 0.000 < 0.001), indicating that emotional deprivation had a significant negative influence on the willingness to withdraw. Therefore, H2c is valid.

#### 4.2.2. Path Analysis of the Influence of Social Trust on the Willingness to Withdraw

The standardized path coefficient of social trust on withdraw intention was 0.142 (t-value = 3.508; *p* = 0.000 < 0.001), indicating that social trust has a significant positive effect on withdrawal intention. Thus, H3 holds.

#### 4.2.3. Path Analysis of the Influence of Relative Deprivation Feeling on Social Trust

The standardized path coefficient of economic deprivation on social trust was −0.416 (t-value = −13.888; *p* = 0.000 < 0.001), indicating that economic deprivation had a significant negative effect on social trust. Thus, H1a is valid. The standardized path coefficient of social deprivation on social trust was −0.329 (t-value = −11.309; *p* = 0.000 < 0.001), indicating that social deprivation had a significant negative influence on social trust. Therefore, H1b is valid. The standardized path coefficient of emotional deprivation on social trust was −0.18 (t-value = −7.119 l *p* = 0.000 < 0.001), indicating that emotional deprivation had a significant negative influence on social trust. Thus, H1c is valid.

#### 4.2.4. Analysis of the Mediating Effect of Social Trust

To verify whether there exists a mediating role of social trust in the path of relative deprivation’s influence on willingness to withdraw, a mediating effects test was conducted using the Bootstrap method in the AMOS software, with 2000 replicate samples and 95% confidence intervals. The results of the analysis are shown in Table 8.

In the mediated path of economic deprivation–social trust–willingness to withdraw, the total effect value was −0.285, the 95% confidence interval was negative and did not contain 0, and the *p*-value was less than 0.05, indicating that the total effect existed. The direct effect value was −0.226, the 95% confidence interval was negative and did not contain 0, and the *p*-value was less than 0.05. The indirect effect value was −0.059, the 95% confidence interval was negative and did not contain 0, and the *p*-value was less than 0.05. Therefore, the indirect effect existed, accounting for 20.7% of the total effect, and H4a is valid.

In the mediated path of social deprivation–social trust–willingness to withdraw, the total effect value was −0.356, the 95% confidence interval was negative and did not contain 0, and the *p*-value was less than 0.05, indicating that the total effect existed. The direct effect value was −0.309, the 95% confidence interval was negative and did not contain 0, and the *p*-value was less than 0.05. The indirect effect value was −0.047, the 95% confidence interval was negative and did not contain 0, and the *p*-value was less than 0.05. Therefore, the indirect effect existed, accounting for 13.2% of the total effect, and H4b is valid.

In the mediated path emotional deprivation–social trust–willingness to withdraw, the total effect value was −0.223, the 95% confidence interval was negative and did not contain 0, and the *p*-value was less than 0.05, indicating that the total effect existed. The direct effect value was −0.198, the 95% confidence interval was negative and did not contain 0, and the *p*-value was less than 0.05. The indirect effect value was −0.026, the 95% confidence interval was negative and did not contain 0, and the *p*-value was less than 0.05. Therefore, the indirect effect existed, accounting for 11.2% of the total effect, and H4c is valid.

#### 4.2.5. Analysis of the Moderating Effect of Urban Integration

(1) Test of the moderating role of urban integration in the relationship between relative deprivation and social trust.

The test of the moderating effect was mainly conducted using multiple cascade regression, mainly involving the construction of two multiple regression models. The second model introduces the independent and moderating variables, in order to determine whether the independent and moderating variables affect the dependent variable and determine the explanatory power of the model; that is, to judge the model R^2^. The third model introduces the independent and moderating variables and their interaction terms. If the regression coefficient of the interaction term is significant and the R^2^ value is significantly higher, it means that the moderating variables have a significant effect (Table 9).

As shown in the table above, Model 1 was a multiple regression model with economic deprivation and urban integration as independent variables and social trust as a dependent variable, while Model 2 was a multiple regression model with economic deprivation, urban integration, and the interaction term economic deprivation × urban integration as independent variables and social trust as a dependent variable. In Model 1, the independent variable economic deprivation had a significant negative effect on social trust (β = −0.600; t = −27.162; *p* < 0.001). In Model 2, the regression coefficient of the interaction term between the independent variable and the moderating variable was 0.036 (t = 1.612; *p* > 0.05), indicating that the interaction term did not have a significant effect on social trust. The R² of Model 1 was 0.370 and that for Model 2 was 0.371, which did not change significantly. Therefore, the moderating variable—urban integration—does not play a significant moderating role in the relationship between the effect of economic deprivation and social trust. Thus, H5a is not valid.

1) Testing the moderating role of urban integration in the relationship between social deprivation and social trust.

As shown in the table above, Model 1 was a multiple regression model with economic deprivation and urban integration as independent variables and social trust as a dependent variable, while Model 2 was a multiple regression model with economic deprivation, urban integration, and the interaction term economic deprivation × urban integration as independent variables and social trust as a dependent variable. In Model 1, the independent variable economic deprivation has a significant negative effect on social trust (β = −0.600; t = −27.162; *p* < 0.001). In model 2, the regression coefficient of the interaction term between the independent variable and the moderating variable was 0.036 (t = 1.612; *p* > 0.05), indicating that the interaction term did not have a significant effect on social trust. The R² of Model 1 is 0.370 and that for Model 2 was 0.371, which did not change significantly. Therefore, it the moderating variable urban integration does not have a significant moderating role in the relationship between the effect of economic deprivation and social trust. Therefore, H5a is not valid (Table 10).

2) Test of the moderating role of urban integration in the relationship between social deprivation and social trust.

**Figure 2:**
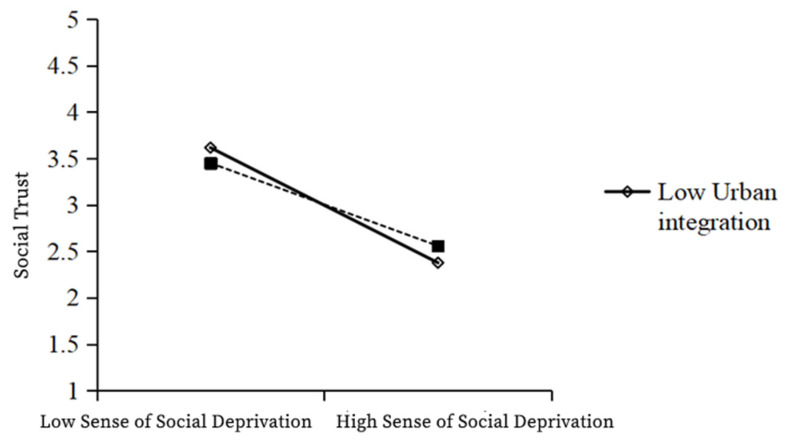
Graph of the moderating effect of urban integration in the relationship between the sense of social deprivation and social trust.

As shown in the table above, Model 1 was a multiple regression model with social deprivation and urban integration as independent variables and social trust as a dependent variable, while Model 2 was a multiple regression model with social deprivation, urban integration, and the interaction term social deprivation × urban integration as independent variables and social trust as a dependent variable. In Model 1, the independent variable social deprivation has a significant negative effect on social trust (β = −0.594; t = −26.378; *p* < 0.001). In Model 2, the regression coefficient of the interaction term between the independent variable and the moderating variable was 0.097 (t = 4.370; *p* < 0.001), indicating that the interaction term had a significant positive effect on social trust. The R² of Model 1 was 0.357, and that for Model 2 was 0.366, which was significantly higher, indicating the enhanced explanatory power of the model. Therefore, the moderating variable—urban integration—has a significant positive moderating effect in the relationship between social deprivation and social trust. In particular, urban integration weakens the negative relationship between social deprivation and social trust, and so, H5b is valid (Table 11, Figure 2).

3) Test of the moderating role of urban integration in the relationship between emotional deprivation and social trust.

**Figure 3:**
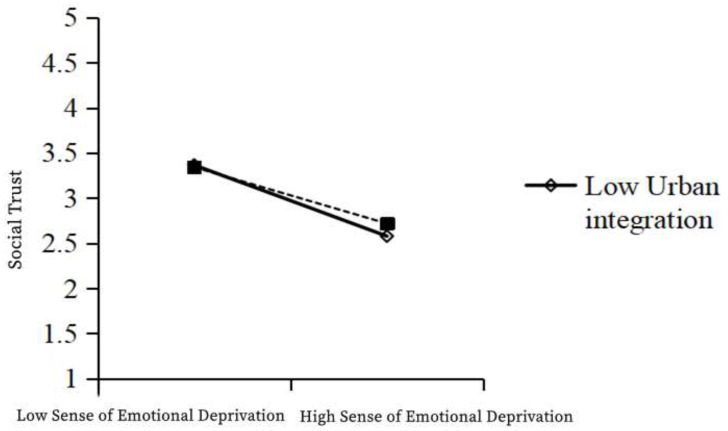
The moderating effect of urban integration on the relationship between emotional deprivation and social trust.

As shown in the table above, Model 1 was a multiple regression model with emotional deprivation and urban integration as independent variables and social trust as a dependent variable, while Model 2 was a multiple regression model with emotional deprivation, urban integration, and the interaction term emotional deprivation × urban integration as independent variables and social trust as a dependent variable. In Model 1, the independent variable emotional deprivation had a significant negative effect on social trust (β = −0.160; t = −18.543; *p* < 0.001). In Model 2, the regression coefficient of the interaction term between the independent variable and the moderating variable was 0.053 (t = 2.148; *p* < 0.001), indicating that the interaction term had a significant positive effect on social trust. The R² of model 1 was 0.221 and that for Model 2 was 0.224, comprising a significant improvement, indicating the enhanced explanatory power of the model. Therefore, it the moderating variable urban integration has a significant positive moderating effect on the relationship between emotional deprivation and social trust. Thus, urban integration weakens the negative relationship between emotional deprivation and social trust, and so, H5c is valid (Table 12, Figure 3).

(2) Test of the moderating role of urban integration in the relationship between relative deprivation and willingness to withdraw.

1) Testing the moderating role of urban integration in the relationship between the sense of economic deprivation and the willingness to withdraw.

**Figure 4:**
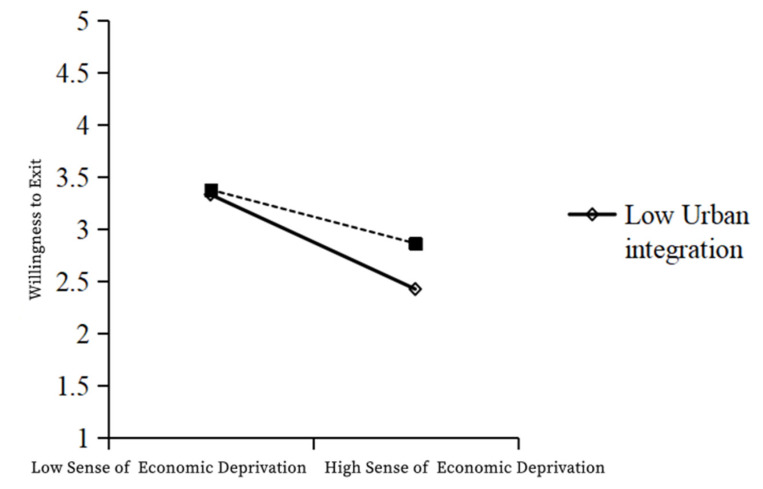
Moderating effect of urban integration between the sense of economic deprivation and willingness to withdraw.

As shown in the table above, Model 1 was a multiple regression model with sense of economic deprivation and urban integration as independent variables and withdrawal intention as a dependent variable, while Model 2 was a multiple regression model with sense of economic deprivation, urban integration, and the interaction term sense of economic deprivation × urban integration as independent variables and withdrawal intention as a dependent variable. In Model 1, the independent variable economic deprivation had a significant negative effect on withdrawal intention (β = −0.455; t = −18.911; *p* < 0.001). In Model 2, the regression coefficient of the interaction term between the independent variable and the moderating variable was 0.126 (t = 5.313; *p* < 0.001), indicating that the interaction term has a significant positive effect on withdrawal intention. The R² of Model 1 was 0.256 and that of Model 2 was 0.271, presenting a significant improvement, indicating the enhanced explanatory power of the model. Therefore, the moderating variable—urban integration—had a significant positive moderating effect on the relationship between economic deprivation and the willingness to withdraw. In particular, urban integration weakens the negative relationship between the feeling of economic deprivation and the willingness to withdraw, and so, H6a is valid (Table 13, Figure 4).

2) Test of the moderating effect of urban integration between the sense of social deprivation and the willingness to withdraw.

**Figure 5:**
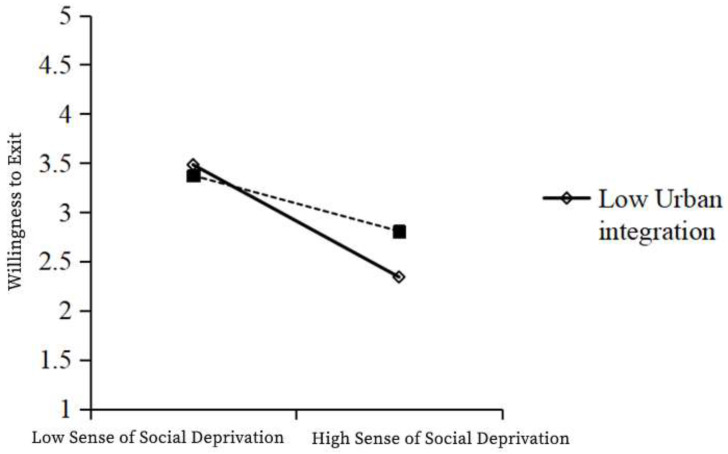
Moderating effect of urban integration between the sense of social deprivation and willingness to withdraw.

As shown in the table above, Model 1 was a multiple regression model with social deprivation and urban integration as independent variables and withdrawal intention as a dependent variable, while Model 2 was a multiple regression model with social deprivation, urban integration, and the interaction term social deprivation × urban integration as independent variables and withdrawal intention as a dependent variable. In Model 1, the independent variable social deprivation had a significant negative effect on withdrawal intention (β = −0.507; t = −21.6; *p* < 0.001). In Model 2, the regression coefficient of the interaction term between the independent variable and the moderating variable was 0.172 (t = 7.523; *p* < 0.001), indicating that the interaction term had a significant positive effect on withdrawal intention. The R² of Model 1 was 0.301 and that of Model 2 was 0.330, presenting a significant improvement and indicating the enhanced explanatory power of the model. Therefore, the moderating variable—urban integration—has a significant positive moderating effect on the relationship between social deprivation and the willingness to withdraw. In particular, urban integration weakens the negative relationship between social deprivation and willingness to withdraw, and so, H6b is valid (Table 14, Figure 5).

3) Test of the moderating role of urban integration between emotional deprivation and willingness to withdraw.

**Figure 6:**
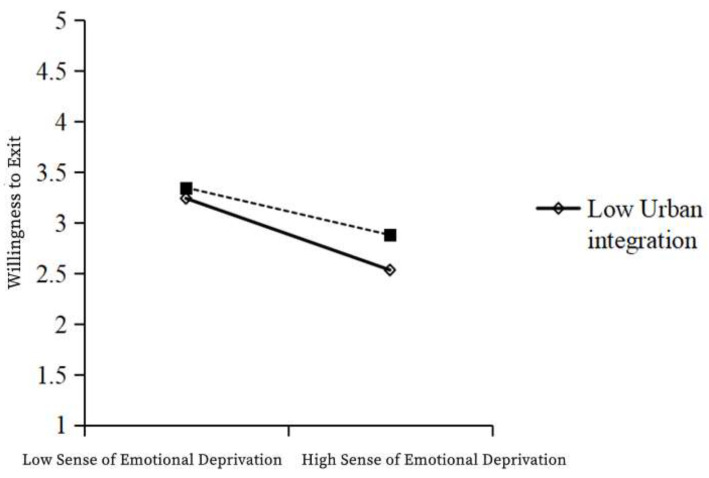
Moderating effect of urban integration between the sense of emotional deprivation and willingness to withdraw.

As shown in the table above, Model 1 was a multiple regression model with emotional deprivation and urban integration as independent variables and withdrawal intention as a dependent variable, while Model 2 was a multiple regression model with emotional deprivation, urban integration, and the interaction term emotional deprivation × urban integration as independent variables and withdrawal intention as a dependent variable. In Model 1, the independent variable emotional deprivation had a significant negative effect on withdrawal intention (β = −0.409; t = −16.429; *p* < 0.001). In Model 2, the regression coefficient of the interaction term between the independent variable and the moderating variable was 0.083 (t = 3.373; *p* < 0.001), indicating that the interaction term has a significant positive effect on withdrawal intention. The R^2^ of Model 1 was 0.214 and that of Model 2 was 0.221, showing a significant improvement, indicating the enhanced explanatory power of the model. Therefore, the moderating variable—urban integration—has a significant positive moderating effect on the relationship between emotional deprivation and willingness to withdraw. In particular, urban integration weakens the negative relationship between emotional deprivation and willingness to withdraw, and so, H6c is valid (Table 15, Figure 6).

## 5. Discussion

Compared with previous studies, this study has more comprehensive considerations. Using the research data of 1320 households in Jinan, we analyzed the interaction and influence of the emotional psychological factors and social environmental factors of the peasants who entered the city on their willingness to withdraw from the homestead. We divided the relative deprivation of farmers into economic deprivation, social deprivation and emotional deprivation innovatively. At the same time, we empirically explored the internal mechanism of the impact of various dimensions of relative deprivation on farmers’ homestead withdrawal and differentiated the intermediary effect of social trust and the regulatory effect of urban integration.

First, we found that relative deprivation has a significant negative effect on the homestead withdrawal behavior of farmers, which inhibits them from withdrawing from their homesteads. Second, social trust was found to play a mediating role in all dimensions of relative deprivation on the homestead withdrawal behavior of farmers. Third, urban integration was found to play a positive moderating role in inhibiting the homestead withdrawal behavior of farmers: the higher the degree of urban integration, the weaker the inhibiting effect of relative deprivation on the withdrawal behavior. Urban integration was also found to play a positive moderating role in inhibiting the effects of social deprivation and emotional deprivation on social trust, such that an increase in the urban integration degree can weaken the influence of emotional deprivation on the homestead withdrawal behavior of farmers.

As a member of the rural collective, the homestead is a source of income. As a necessary place for rural collective members to live and use without compensation for an indefinite period of time, the homestead guarantees the survival needs of members of the collective. Therefore, the homestead also assumes part of the function of social security [12]. However, when farmers move to the city, it is difficult for them to get a home and to enjoy the same treatment as urban residents, in terms of employment, pension, education, and medical care, such that withdrawal from their homesteads may induce a loss of social security function rights and interests [8]. Therefore, the implementation of urban farmer homestead withdrawal may generate a strong sense of social deprivation, considering the loss of their social security function and rights. This sense of social deprivation that inhibits their willingness to withdraw from their homestead bases. At the same time, the local society has endowed the homestead with the function of emotional inheritance, carrying the farmer’s love for the land and emotional attachment [69]. When farmers are faced with the problem of withdrawing from their homestead base, it may bring about an expectation of loss of emotional functional rights and interests, thus generating a strong sense of emotional deprivation, which will inhibit their homestead withdrawal behavior. Although relative deprivation is unavoidable, people can resolve and prevent it in certain ways. Improving farmers’ social trust is one of the effective ways to alleviate the sense of relative deprivation.

Regarding H1, these results supported H1a–H1c. The results are consistent with the results of Pierce, J.L. et al. (2001) [70], Niu, X. F. et al. (2021) [71] and Morewedge, C. K. et al. (2009) [72]. First, the homestead base not only facilitates agricultural production activities and the development of yard economy for farmers but, also, with the state’s emphasis on the usufruct rights of homestead bases, the economic property attributes of the homestead bases are increasingly obvious. Therefore, this will raise the expectation regarding the economic functional value of the homestead, and the expected loss of economic functional rights and interests brought by the withdrawal of the homestead will generate a strong sense of economic deprivation which, in turn, will inhibit the willingness to withdraw from the homestead [38,72].

Regarding H2, these results supported H2a–H2c. The results are consistent with the results of Hooghe, M. et al. (2017) [73] and Fischer, J.A.V. et al. (2013) [74].Generally speaking, individuals who are successful in socio-economic life are more likely to trust state institutions [73]. Then, relative to the rural residents who are in a disadvantaged position, the generation of a sense of relative deprivation affects their degree of trust in social, organizational, power, management, and governance systems [74]. Although a sense of relative deprivation is unavoidable, it can be resolved and prevented in certain ways, providing a means to enhance the social trust of farmers. Social trust is one of the means with which the sense of relative deprivation can be hedged.

Regarding H3, these results supported H3. The results are consistent with the results of Beaudoin, C.E. et al. (2004) [45], Meng, T. et al. (2014) [75] and Jiang, J. et al. (2021) [47]. (1) Good social trust can enhance their courage to withdraw from their homestead bases and eliminate their fear of the unknown risks that may be brought about by the homestead reform [75]. In turn, this can enhance their willingness to withdraw from their homestead bases [35]. (2) The higher the trust of farmers in village cadres or councils, the stronger the guarantee mechanism of village cadres or councils and the more confidence they have in the household registration commitment, compensation commitment, and policy commitment being true and credible, which plays an important role in overcoming the psychological “uncertainty in homestead withdrawal” of farmers. Thus, farmers will be motivated to choose to withdraw from their homestead bases. (3) It is believed that judicial, administrative, and legislative bodies will protect the legal rights and interests of the rural-to-urban migrant workers and provide legal support for them to live and work in the city. In this way, the social trust of the urban migrant population can be enhanced, which, in turn, will enhance the willingness of farm households to withdraw from their homesteads.

Regarding H4, these results supported H4a–H4c. The results are consistent with the results of Shen, S. et al. (2022) [47] and Xu, Z. et al. (2020) [51]. The specific impact paths of relative deprivation and governmental trust on the willingness of farmers to withdraw can be described as follows: (1) The negative emotional reactions and cognitive changes derived from relative deprivation, as well as the frustration generated by social comparison, lead some members of society with an avoidance mentality to adopt outward attribution. This will, to a certain extent, reduce their trust in the government and promote their recourse to extra-institutional means of benefit protection, which has a negative impact on their willingness to withdraw from their homestead [76]. (2) The local government is the main government organization related to the distribution of social benefits and the implementation of policies and measures for rural residents [77]. A sense of relative deprivation will lead to dissatisfaction in the social living standard and social status of rural residents, which will lead them to question the performance of the local government or the competence of its staff [48]. This will affect the trust of rural residents in the grassroots government, thus affecting the willingness of rural households to withdraw.

Regarding H5, these results supported H5b and H5c, but not supported H5a. Regarding H5a, the results are not consistent with the results of Chen, Z. et al. (2019) [32]. Theoretically, urban integration can alleviate the economic pressure or reduce the various forms of material deprivation of retiring farmers by providing stable employment, a livable environment and complete public services, thus weakening the negative influence of the sense of economic deprivation on farmers’ homestead withdrawal behavior. However, from the current data, there is no significant impact. It may be because: migrant workers only consider the city as a place to work, and do not consider themselves as part of the city, and they do not obtain the corresponding emotional support and sense of value, which will reduce their willingness to integrate into the local city. This results in an involutional identity of migrant workers, which increases the social distance between migrant workers and the city, thus causing the migrant worker group to voluntarily choose to form their own community network and create a deep separation from the urban society, thus affecting their trust level in the city. [32]. Regarding H5b and H5c, the results are consistent with the results of Sun, X. et al. (2022) [31] and Wang, W.W. et al. (2012) [34]. Firstly, urban integration can expand an individual’s options to participate in political and community activities by giving them various opportunities for social participation, thus enhancing their social rights and abilities through participation in social interactions and, consequently, weakening the negative influence of the sense of social deprivation on the homestead withdrawal behavior of farmers [31]. Secondly, urban integration involves achieving integration into urban culture and identifying oneself as an “urbanite”. Having a psychologically comfortable urban life can only be achieved when the psychological integration of urban farmers is completed [34]. Psychological integration will motivate farmers to choose to settle in the city, significantly weakening their cultural and emotional attachment to the homestead, such that they will be more willing to give up their old homes in the countryside and better realize their settlement in the city using the property gains from the withdrawal from their homestead. In other words, psychological integration affects the negative influence on the homestead withdrawal behavior of farmers by weakening their sense of emotional deprivation.

Regarding H6, these results supported H6. The results are consistent with the results of Xu, D. et al. (2020) [78] and Xu, Y. et al. (2021) [51]. In general, rural societies are acquaintance-oriented societies, while urban societies are stranger-oriented societies. As urban farmers leave the society of acquaintances and come to the city to make a living, the anonymity and fickleness of human interactions are strong, and it becomes more and more difficult to establish social trust [79]. The reason for the social distrust of urban migrant farmers is that they are disadvantaged in the distribution of social resources, due to institutional and policy constraints [78]; that is, therefore, it can be hypothesized that high-quality urban integration can enhance social identity and sense of belonging by improving the quality of life, forming solidarity and stable values, and thus weakening the negative effects of emotional deprivation.

## 6. Conclusions

In the context of the accelerated development of new urbanization, exploring and establishing a mechanism for the paid withdrawal from rural residential bases is considered a general trend for rural residential base system reform in the future, which is of great significance to revitalize the inefficiently utilized land resources in rural areas, activate the dormant land assets in rural areas, increase the property income of farmers, optimize the land structure and spatial layout of urban and rural areas, and promote rural revitalization. The following aspects should be considered in such processes.

(1) The government should pay attention to and helping farmers to rationalize the property rights of residential bases. On one hand, although the legal level clearly defines that the ownership of residential bases belongs to the collective, most farmers have conducted a de facto “blurring” of the concept of “collective”, such that it is necessary to further clarify and standardize the meaning and scope of “collective. For this reason, publicity and popularization should be carried out by building an information platform regarding residential base policy, in order to gradually weaken the misperception of “possession means ownership” in the traditional thinking of farmers. On the other hand, clarifying property rights relationships and the registration of rights is fundamental. Government should speed up the process of issuing real estate property rights certificates, as well as further deepening the knowledge and understanding of farmers in the process of confirming and issuing certificates.

(2) Weakening the sense of relative deprivation in farmers. First, we need to accelerate the improvement of the market price formation mechanism for rural residential bases. In order to achieve the reasonableness and fairness of the withdrawal compensation and value-added income, the withdrawal cost of farmers should be shared through realization of the property value of residential bases, which will help to weaken their sense of economic deprivation. Second, focus should be placed on the livelihood and development of farmers after their withdrawal from residential bases; improving social acceptance; and achieving fair rights, fair opportunities, and fair distribution in the construction of social security, in order to alleviate the worries of farmers who have withdrawn from their residential bases and weaken their sense of social deprivation. The third is to improve the ability of migrant workers to integrate into society. Governments should focus on designing inclusive development policies to promote the social integration of urban farmers. Link government performance with inclusive governance to improve the government’s incentive for inclusive governance. At the same time, encourage social organizations to synergize with government departments and play a complementary function to ensure the equalization of public services. Attention should also be paid to building a socially inclusive environment with equal opportunities and shared outcomes, eliminating various forms of social exclusion and weakening their sense of emotional deprivation.

(3) Improve and enhance the level of social trust of farming households. Given that social trust has a good external effect, society should actively advocate the concept of equal pay for equal work and equal rights in the same city, guarantee the citizenship of the rural-to-urban migrant workers, and improve the trust level in the rural-to-urban migrant workers. The government, society, and enterprises are the protagonists of social trust building, and these three parties need to coordinate and cooperate in order to effectively promote the social integration of the rural-to-urban migrant workers. The government plays the role of coordination, supervision, and management, with a focus on balancing the social rights of the rural-to-urban migrant workers and granting them equal citizenship rights when designing policies. Society should enhance the city’s inclusive and pluralistic character, adopting an accepting attitude toward the rural-to-urban migrant workers and improving urban governance. Employers should treat foreigners equally, take social responsibility, provide vocational training and benefits to foreigners, and promote professional integration. Only in this way can urban society achieve long-lasting peace and stability, in the process of which the rural-to-urban migrant workers can truly achieve welfare growth and social integration.

First, village and township cadres should effectively perform their duties, reach out to the masses, and respond to needs of farmers and win their trust. Second, construction of residential base withdrawal mechanisms should be conducted differently, according to the local conditions and orderly promotion of residential base withdrawal. Due to the large differences in the locations of homesteads and farm buildings, different family planning patterns, and different levels of social trust, in order to avoid various social risks and contradictions in the process of homestead withdrawal, it is necessary to develop differentiated policies which are applicable to different groups of farmers and avoid one-sided “one-size-fits-all” practices, which may cause adverse social consequences. Third, the social integration capacity of farmers should be improved, through designing inclusive development policies to promote the social integration of farmers into cities. In this context, the performance of the government should be integrated in an inclusive manner to improve the government’s incentive for inclusive governance; second, social organizations should be encouraged to synergize with government departments, playing a complementary function to ensure the equality of public services; and, finally, a socially inclusive environment with equal opportunities and shared results to eliminate various forms of social exclusion should be built up, thus innovating social participation and improving social empowerment.

## Figures and Tables

**Table 1:** Relative deprivation evaluation index.

Dimension	Indicators	Average Value	Standard Deviation
Sense of economic deprivation	1. I think it is inconvenient for agricultural production after withdrawing from the house base	3.46	1.228
	2. I think it costs too much to buy a new house after withdrawing from the house site	3.38	1.275
	3. I think the cost of living increases a lot after withdrawing from the residential land	3.35	1.184
Sense of social deprivation	4. I think employment is a problem after withdrawing from the homestead	3.61	1.119
	5. I think it’s a problem to retire after withdrawing from the homestead	3.69	1.114
	6. I don’t think I can enjoy the same urban treatment after withdrawing from the residential base	3.71	1.065
Sense of emotional deprivation	7. I feel that I don’t have a sense of belonging in the new environment after withdrawing from my homestead	3.12	1.266
	8. I feel that after I quit my homestead, I will feel lonely because I have fewer neighbors to interact with	3.11	1.321
	9. I think I will stay in my original village life after I quit the house site	3.13	1.236

**Table 2:** Social trust evaluation index.

Dimension	Indicators	Average Value	Standard Deviation
Government Trust	1. I believe the government can protect us wage earners	2.23	1.101
	2. If you are infringed by the rights and interests, find the government can be used	2.43	1.18
Legal Trust	3. I believe that my rights and interests can be protected through legal means	2.46	1.015
	4. I believe that the law does not protect only the powerful and rich people	2.45	1.097
Urban Environmental Trust	5. There are still more good people in the city	2.44	1.074
	6. I like the urban environment	2.49	1.082
Social Equity Trust	7. I believe that as long as I work hard, working people can also be very successful	2.43	1.149
	8. I believe that this society is still very fair	2.38	1.098

**Table 3:** Urban integration evaluation index.

Dimension	Indicators	Average Value	Standard Deviation
Economic Integration	1. I have a satisfactory employment situation	3.3	1.165
	2. I have the financial ability to accept city price levels	3.29	1.182
	3. I have satisfactory living conditions	3.33	1.241
Social Inclusion	4. I get along with local people	3.35	1.265
	5. I am willing to solve problems with other residents if there are problems affecting the whole neighborhood	3.34	1.207
	6. I understand the difference in social rules between urban and rural areas	3.33	1.246
Psychological integration	7. I have urban identity	3.31	1.211
	8. I wish to contribute to the development of the city	3.3	1.146
	9. I feel at home locally	3.32	1.142

**Table 4:** Homestead withdrawal intention evaluation index.

Dimensional	Indicators	Average Value	Standard Deviation
Willingness to withdraw	1. I am willing to exchange my homestead for a government-unified resettlement house	2.35	0.978
	2. I am willing to exchange my homestead for a commercial house of the same size	2.37	0.987
	3. I am willing to exchange my homestead for financial compensation	2.41	0.962

**Table 5:** Descriptive Statistics of Samples.

Subjects’ General Information	Category	Frequency	Percentage
Gender	Male	621	47
	Female	699	53
Age	19–30 years old	208	15.8
	31–40 years old	317	24
	41–50 years old	344	26.1
	51–60 years old	238	18
	61 years old and above	213	16.1
Current Residence Address	Lixia District	251	19
	Licheng District	331	25.1
	Changqing District	199	15.1
	Samuel District	212	16.1
	Zhangqiu District	327	24.8
Monthly income	Under 3500 RMB	103	7.8
	3500–4500 RMB	331	25.1
	4500–5500 RMB	449	34
	5500–6500 RMB	265	20.1
	6500 RMB and above	172	13
Academic qualifications	Undergraduate	106	8
	College	212	16.1
	High school or junior college	383	29
	Junior High School	423	32
	Elementary school and below	196	14.8
How did you get your current job?	Apply for the job yourself	647	49
	Referral from friends, relatives, or acquaintances	251	19
	Introduction of intermediary organizations	422	32
Your current housing type	Unit allocation	199	15.1
	Government low-cost housing	133	10.1
	Individual Rental	727	55.1
	Individual purchase	261	19.8
Work Unit	Manufacturing	199	15.1
	Transportation, storage, and postal industry	195	14.8
	Construction	302	22.9
	Accommodation and Catering	245	18.6
	Wholesale and retail trade	168	12.7
	Residential service repair industry	211	16

**Table 6:** Factor analysis results.

Variables	Measurement Topics	Estimate	SE	CR	*p*	CR	AVE
Sense of economic deprivation	A1	0.831				0.851	0.656
	A2	0.83	0.032	32.065	***		
	A3	0.767	0.03	29.593	***		
Sense of social deprivation	A4	0.885				0.898	0.745
	A5	0.887	0.024	42.419	***		
	A6	0.816	0.023	37.448	***		
Sense of emotional deprivation	A7	0.858				0.906	0.762
	A8	0.913	0.026	42.105	***		
	A9	0.846	0.025	38.421	***		
Social Trust	B1	0.886				0.931	0.628
	B2	0.8	0.025	38.1	***		
	B3	0.71	0.024	31.228	***		
	B4	0.748	0.025	33.879	***		
	B5	0.791	0.023	37.348	***		
	B6	0.739	0.025	33.273	***		
	B7	0.771	0.025	35.657	***		
	B8	0.877	0.022	45.589	***		
City Integration	C1	0.818				0.954	0.697
	C2	0.826	0.028	35.988	***		
	C3	0.847	0.029	37.383	***		
	C4	0.856	0.03	38.005	***		
	C5	0.839	0.029	36.876	***		
	C6	0.841	0.03	37.008	***		
	C7	0.844	0.029	37.18	***		
	C8	0.824	0.028	35.868	***		
	C9	0.818	0.028	35.46	***		
Willingness to withdraw	D1	0.818				0.836	0.629
	D2	0.796	0.033	29.406	***		
	D3	0.765	0.033	28.315	***		

Note: ***, *p* < 0.001, CR, critical ratio (i.e., the t-value corresponding to each item loading); SE, standard error.

**Table 7:** Path analysis results.

Hypothetical Path			Estimate	SE	CR	*p*
Social Trust	<--	Sense of economic deprivation	−0.416	0.032	−13.888	***
Social Trust	<--	Sense of social deprivation	−0.329	0.033	−11.309	***
Social Trust	<--	Sense of emotional deprivation	−0.18	0.024	−7.119	***
Willingness to withdraw	<--	Sense of economic deprivation	−0.226	0.034	−5.92	***
Willingness to withdraw	<--	Sense of social deprivation	−0.309	0.033	−8.539	***
Willingness to withdraw	<--	Sense of emotional deprivation	−0.198	0.023	−6.557	***
Willingness to withdraw	<--	Social Trust	0.142	0.033	3.508	***

Note: ***, *p* < 0.001.

**Table 8:** Bootstrap method mediating effects test results.

Effect Path	Effect Type	Effect Value	SE	95% Confidence Interval		*p*	Effectiveness Ratio
				Lower Interval	Upper Interval		
Sense of economic deprivation–Social trust–Willingness to withdraw	Total effect	−0.285	0.034	−0.35	−0.218	0.001 **	-
	Direct effect	−0.226	0.046	−0.316	−0.133	0.001 **	79.3%
	Indirect effects	−0.059	0.027	−0.111	−0.006	0.027 *	20.7%
Sense of social deprivation–Social trust–Willingness to withdraw	Total effect	−0.356	0.034	−0.427	−0.29	0.001 **	-
	Direct effect	−0.309	0.041	−0.392	−0.227	0.001 **	86.8%
	Indirect effects	−0.047	0.021	−0.088	−0.005	0.027 *	13.2%
Sense of emotional deprivation–Social trust–Willingness to withdraw	Total effect	−0.223	0.035	−0.291	−0.158	0.001 **	-
	Direct effect	−0.198	0.037	−0.271	−0.125	0.001 **	88.8%
	Indirect effects	−0.026	0.011	−0.047	−0.003	0.023 *	11.2%

Note: *, *p* < 0.05; **, *p* < 0.01.

**Table 9:** The moderating effect of urban integration between perceived economic deprivation and social trust.

Variables	Model 1		Model 2	
	Β	t	β	t
Sense of economic deprivation	−0.600 ***	−27.162	−0.600 ***	−27.15
Urban Integration	0.044 *	1.988	0.039 *	1.741
Sense of economic deprivation × Urban integration	-	-	0.036 *	1.612
R²	0.370		0.371	
Adjusted R²	0.369		0.370	
ΔR²	-		0.001	
F	387.146 ***		259.277 ***	

Note: ***, *p* < 0.001; *, *p* < 0.05.

**Table 10:** Test of the moderating effect of urban integration between perception of social deprivation and social trust.

Variables	Model 1		Model 2	
	β	t	β	t
Sense of economic deprivation	−0.600 ***	−27.162	−0.600 ***	−27.15
Urban Integration	0.044 *	1.988	0.039 *	1.741
Sense of economic deprivation × Urban integration	-	-	0.036	1.612
R²	0.370		0.371	
Adjusted R²	0.369		0.370	
ΔR²	-		0.001	
F	387.146 ***		259.277 ***	

Note: ***, *p* < 0.001; *, *p* < 0.05.

**Table 11:** Test of the moderating effect of urban integration between perception of social deprivation and social trust.

Variables	Model 1		Model 2	
	β	t	β	t
Sense of social deprivation	−0.594 ***	−26.378	−0.593 ***	−26.522
Urban Integration	0.020	0.876	0.004 **	0.185
Social deprivation × Urban integration	-	-	0.097 ***	4.370
R²	0.357		0.366	
Adjusted R²	0.356		0.365	
ΔR²	-		0.009	
F	365.784 ***		253.571 ***	

Note: *** *p* < 0.001, ** *p* < 0.01.

**Table 12:** Test of the moderating effect of urban integration on the relationship between emotional deprivation and social trust.

Variables	Model 1		Model 2	
	β	t	β	t
Sense of emotional deprivation	−0.460 ***	−18.543	−0.458 ***	−18.481
Urban Integration	0.041	1.647	0.033 *	1.301
Emotional deprivation × Urban integration	-	-	0.053 *	2.148
R²	0.221		0.224	
Adjusted R²	0.220		0.222	
ΔR²	-		0.003	
F	186.690 ***		126.339 ***	

Note: ***, *p* < 0.001; *, *p* < 0.05.

**Table 13:** The moderating effect of urban integration on the relationship between economic deprivation and willingness to withdraw.

Variables	Model 1		Model 2	
	β	t	β	t
Sense of economic deprivation	−0.455 ***	−18.911	−0.453 ***	−19.021
Urban Integration	0.164 ***	6.835	0.146 ***	6.082
Sense of economic deprivation × Urban integration	-	-	0.126 ***	5.313
R²	0.256		0.271	
Adjusted R²	0.254		0.270	
ΔR²	-		0.016	
F	226.039 ***		163.217 ***	

Note: ***, *p* < 0.001.

**Table 14:** The moderating effect of urban integration on the relationship between social deprivation and willingness to withdraw.

Variables	Model 1		Model 2	
	β	t	β	t
Sense of social deprivation	−0.507 ***	−21.6	−0.505 ***	−21.99
Urban Integration	0.135 ***	5.755	0.108 ***	4.623
Sense of social deprivation × Urban integration			0.172 ***	7.523
R²	0.301		0.330	
Adjusted R²	0.300		0.328	
ΔR²	-		0.029	
F	283.573 ***		215.895 ***	

Note: ***, *p* < 0.001.

**Table 15:** Test of the moderating effect of urban integration on the relationship between emotional deprivation and willingness to withdraw.

Variables	Model 1		Model 2	
	β	t	β	t
Sense of emotional deprivation	−0.409 ***	−16.429	−0.406 ***	−16.364
Urban Integration	0.15 ***	6.013	0.137 ***	5.449
Emotional deprivation × Urban integration	-	-	0.083 **	3.373
R²	0.214		0.221	
Adjusted R²	0.213		0.219	
ΔR²	-		0.007	
F	179.702 ***		124.537 ***	

Note: ***, *p* < 0.001; **, *p* < 0.01.

## Data Availability

Not applicable.

## References

[1] Liu R., Yu C., Jiang J., Huang Z., Jiang Y. (2020). Farmer differentiation, generational differences and farmers’ behaviors to withdraw from rural homesteads: Evidence from Chengdu, China. Habitat Int..

[2] State Statistical Bureau Monitoring and Investigation Report of Migrant Workers in 2021. www.stats.gov.cn/xxgk/sjfb/zxfb2020/202204/t20220429_1830139.html.

[3] State Statistical Bureau Statistical Bulletin of National Economic and Social Development in 2021. http://www.stats.gov.cn/xxgk/sjfb/zxfb2020/202202/t20220228_1827971.html.

[4] Yuan Z., Fu C., Kong S., Du J., Li W. (2022). Citizenship Ability, Homestead Utility, and Rural Homestead Transfer of “Amphibious” Farmers. Sustainability.

[5] National Agricultural and Rural Sector.Notice on the Active and Steady Development of Rural Idle House Bases and Idle Residential Inventory and Utilization. http://www.moa.gov.cn/nybgb/2019/201910/202001/t20200109_6334695.htm.

[6] Liang F., Wang Z., Lin S.-H. (2022). Can Land Policy Promote Farmers' Subjective Well-Being? A Study on Withdrawal from Rural Homesteads in Jinjiang, China. Int. J. Environ. Res. Public Health.

[7] Han W.L., Liu L. (2020). Tenure awareness, resource endowment, and willingness to quit the homestead. Agric. Econ. Issues.

[8] Chen H., Zhao L., Zhao Z. (2017). Influencing factors of farmers' willingness to withdraw from rural homesteads: A survey in Zhejiang, China. Land Use Policy.

[9] Liu R., Jiang J., Yu C., Rodenbiker J., Jiang Y. (2021). The endowment effect accompanying villagers’ withdrawal from rural homesteads: Field evidence from Chengdu, China. Land Use Policy.

[10] Liang F., Lin C., Lin S.H. (2022). Farmers’ livelihood, risk expectations, and homestead withdrawal policy: Evidence on JinJiang pilot of China. Int. J. Strateg. Prop. Manag..

[11] Lu X., Peng W., Huang X., Fu Q., Zhang Q. (2020). Homestead management in China from the “separation of two rights” to the "separation of three rights”: Visualization and analysis of hot topics and trends by mapping knowledge domains of academic papers in China National Knowledge Infrastructure (CNKI). Land Use Policy.

[12] Liu R. (2022). Incomplete Urbanization and the Trans-Local Rural-Urban Gradient in China: From a Perspective of New Economics of Labor Migration. Land.

[13] Dong G., Zhang W., Xu X., Jia K. (2021). Multi-Dimensional Feature Recognition and Policy Implications of Rural Human-Land Relationships in China. Land.

[14] Dong G., Ge Y., Cao H., Zhai R. (2022). Withdrawal and Transformation of Rural Homesteads in Traditional Agricultural Areas of China Based on Supply-Demand Balance Analysis. Front. Environ. Sci..

[15] Cao Q., Sarker M.N.I., Sun J. (2019). Model of the influencing factors of the withdrawal from rural homesteads in China: Application of grounded theory method. Land Use Policy.

[16] Qin W., Xu L., Wu S., Shao H. (2021). Income, Relative Deprivation and the Self-Rated Health of Older People in Urban and Rural China. Front. Public Health.

[17] Lu P., Yu M., Hu Y. (2020). Contradictions in and improvements to urban and rural residents' housing rights in China's urbanization process. Habitat Int..

[18] Jin L. (2016). Migration, Relative Deprivation, and Psychological Well-Being in China. Am. Behav. Sci..

[19] Kafle K., Benfica R., Winters P. (2020). Does relative deprivation induce migration? Evidence from Sub-Saharan Africa. Am. J. Agric. Econ..

[20] Visser M., Gesthuizen M., Scheepers P. (2014). The Impact of Macro-Economic Circumstances and Social Protection Expenditure on Economic Deprivation in 25 European Countries, 2007-2011. Soc. Indic. Res..

[21] Ayala L., Jurado A., Perez-Mayo J. (2021). Multidimensional deprivation in heterogeneous rural areas: Spain after the economic crisis. Reg. Stud..

[22] Hannum E., Hu W., Park A. (2019). Home, School, and Community Deprivations: A Multi-Context Approach to Childhood Poverty in China. J. Contemp. China.

[23] Yuan Y., Xu M., Cao X., Liu S. (2018). Exploring urban-rural disparity of the multiple deprivation index in Guangzhou City from 2000 to 2010. Cities.

[24] Zhang L., Fan W. (2020). Rural Homesteads Withdrawal and Urban Housing Market: A Pilot Study in China. Emerg. Mark. Financ. Trade.

[25] Wu Y., Mo Z., Peng Y., Skitmore M. (2018). Market-driven land nationalization in China: A new system for the capitalization of rural homesteads. Land Use Policy.

[26] Gao J., Song G., Liu S. (2022). Factors influencing farmers' willingness and behavior choices to withdraw from rural homesteads in China. Growth Chang..

[27] Huang X., Li H., Zhang X., Zhang X. (2018). Land use policy as an instrument of rural resilience—The case of land withdrawal mechanism for rural homesteads in China. Ecol. Indic..

[28] Kong X., Liu Y., Jiang P., Tian Y., Zou Y. (2018). A novel framework for rural homestead land transfer under collective ownership in China. Land Use Policy.

[29] Liu R., Jia Y. (2021). Resilience and Circularity: Revisiting the Role of Urban Village in Rural-Urban Migration in Beijing, China. Land.

[30] Zou J., Deng X. (2022). To inhibit or to promote: How does the digital economy affect urban migrant integration in China?. Technol. Forecast. Soc. Chang..

[31] Sun X., Chen J., Xie S. (2022). Becoming Urban Citizens: A Three-Phase Perspective on the Social Integration of Rural-Urban Migrants in China. Int. J. Environ. Res. Public Health.

[32] Chen Z. (2019). How the choice of reference group matters: Economic integration of rural-to-urban migrants in China. J. Ethn. Migr. Stud..

[33] Wang M., Ning Y. (2016). The Social Integration of Migrants in Shanghai's Urban Villages. China Rev. Interdiscip. J. Greater China.

[34] Wang W.W., Fan C.C. (2012). Migrant Workers’ Integration in Urban China: Experiences in Employment, Social Adaptation, and Self-Identity. Eurasian Geogr. Econ..

[35] Huhe N., Chen J., Tang M. (2015). Social trust and grassroots governance in rural China. Soc. Sci. Res..

[36] Li F., Loyalka P., Yi H., Shi Y., Johnson N., Rozelle S. (2018). Ability tracking and social trust in China's rural secondary school system. Sch. Eff. Sch. Improv..

[37] Chen D., Liu X., Wang C. (2016). Social Trust and Bank Loan Financing: Evidence from China. Abacus-A J. Account. Financ. Bus. Stud..

[38] Lyon F., Porter G. (2009). Market institutions, trust and norms: Exploring moral economies in Nigerian food systems. Camb. J. Econ..

[39] Kim P.H., Li M. (2014). Seeking Assurances When Taking Action: Legal Systems, Social Trust, and Starting Businesses in Emerging Economies. Organ. Stud..

[40] Rapp C. (2016). Moral opinion polarization and the erosion of trust. Soc. Sci. Res..

[41] Su K., Wu J., Yan Y., Zhang Z., Yang Q. (2022). The Functional Value Evolution of Rural Homesteads in Different Types of Villages: Evidence from a Chinese Traditional Agricultural Village and Homestay Village. Land.

[42] Liu Q., Pan H. (2020). Investigation on Life Satisfaction of Rural-to-Urban Migrant Workers in China: A Moderated Mediation Model. Int. J. Environ. Res. Public Health.

[43] Huhe N. (2014). Understanding the Multilevel Foundation of Social Trust in Rural China: Evidence from the China General Social Survey. Soc. Sci. Q..

[44] Cho J. (2014). Will Social Media Use Reduce Relative Deprivation? Systematic Analysis of Social Capital's Mediating Effects of Connecting Social Media Use with Relative Deprivation. Int. J. Commun..

[45] Beaudoin C.E., Thorson E. (2004). Social capital in rural and urban communities: Testing differences in media effects and models. J. Mass Commun. Q..

[46] Jiang J., Li Q., Kang R., Wang P. (2020). Social Trust and Health: A Perspective of Urban-Rural Comparison in China. Appl. Res. Qual. Life.

[47] Shen S., Quan Q. (2022). Rural tourism development perceived by involved local farmers: Evidences from Gaochun County of China. J. Tour. Cult. Chang..

[48] Yao Y., Lu S., Wang H. (2021). Relative Deprivation and Farmers’ Willingness to Participate in Village Governance: Evidence from Land Expropriation in Rural China. Asian Surv..

[49] Ma Y., Koondhar M.A., Liu S., Wang H., Kong R. (2020). Perceived Value Influencing the Household Waste Sorting Behaviors in Rural China. Int. J. Environ. Res. Public Health.

[50] Li F., Sun Y., Zhang Y., Zhang W. (2022). Research on Multi-Dimensional Influencing Factors Regarding the Perceived Social Integration of New Urban Immigrants: An HLM Analysis Based on Data from 58 Large- and Medium-Sized Cities in China. Int. J. Environ. Res. Public Health.

[51] Xu Y., Li B., Huang X. (2021). Outsiders to urban-centric growth: The dual social exclusion of migrant tenant farmers in China. Third World Q..

[52] James S.C. The Foundations of Social Theory, Fang D. (1992). , Translator.

[53] Fukuyama F., Bangli L., Shengli W. (2002). The Great Schism: Human Nature and the Reconstruction of Social Order.

[54] Huang X. (2020). The Chinese Dream: Hukou, Social Mobility, and Trust in Government. Soc. Sci. Q..

[55] Smith H.J., Pettigrew T.F., Pippin G.M., Bialosiewicz S. (2012). Relative Deprivation: A Theoretical and Meta-Analytic Review. Personal. Soc. Psychol. Rev..

[56] Osborne D., Smith H.J., Huo Y.J. (2012). More Than a Feeling: Discrete Emotions Mediate the Relationship Between Relative Deprivation and Reactions to Workplace Furloughs. Personal. Soc. Psychol. Bull..

[57] Xiong M., Ye Y.R. (2016). Relative deprivation: Concept, measurement, influencing factors and role. Adv. Psychol. Sci..

[58] Lee Y. (2021). Government for Leaving No One Behind: Social Equity in Public Administration and Trust in Government. Sage Open.

[59] Vitale D. (2018). A Trust Network Model for Social Rights Fulfilment. Oxf. J. Leg. Stud..

[60] Sun W., Wang X. (2012). Do government actions affect social trust? Cross-city evidence in China. Soc. Sci. J..

[61] Nikitas A., Avineri E., Parkhurst G. (2018). Understanding the public acceptability of road pricing and the roles of older age, social norms, pro-social values and trust for urban policy-making: The case of Bristol. Cities.

[62] Li H.B., Zhang Y.J. (2020). The impact of urban residential welfare of migrant workers on their urban integration—An empirical analysis based on structural equation modeling. Urban Dev. Res..

[63] Tian M., Bao J.L. (2014). A comparative study of migrant urban integration with principal component analysis. Hum. Grography.

[64] Zhao Q. (2015). Residential spatial differentiation and its impact on urban integration of suburban landless farmers—Based on research data from Fenggang County, Guizhou Province. Agric. Econ. Issues.

[65] Kuang F.Y., Chen M.K. (2021). The influence of risk expectation and livelihood capital on farmers’ willingness to quit their homesteads and their intergenerational differences—Based on 456 farmers' survey data in Jiangxi Province. J. Agric. For. Econ. Manag..

[66] Huang Y.X. (2021). The way to solve the problem of reforming the rural house base system[J]. Agric. Econ. Issues.

[67] Peng C.H. (2013). Influence of farmers’ home base property rights perception status on their willingness to quit their home bases--an empirical analysis based on a questionnaire survey of 1413 farm households in six counties of Anhui Province. China Rural. Obs..

[68] Jinan Bureau of Statistics Statistical Bulletin of National Economic and Social Development of Jinan City in 2021.2022-03-04. http://jntj.jinan.gov.cn/art/2022/3/4/art_18254_4745381.html.

[69] Li B., Mi Z., Zhang Z. (2020). Willingness of the New Generation of Farmers to Participate in Rural Tourism: The Role of Perceived Impacts and Sense of Place. Sustainability.

[70] Pierce J.L., Kostova T., Dirks K.T. (2001). Toward a theory of psychological ownership in organizations. Acad. Manag. Rev..

[71] Niu X.F., Yang Y.Z. (2021). Measurement and influencing factors of Endowment Effect of Homestead. Econ. Problems.

[72] Morewedge C.K., Shu L.L., Gilbert D.T., Wilson T.D. (2009). Bad riddance or good rubbish? Ownership and not loss aversion causes the endowment effect. J. Exp. Soc. Psychol..

[73] Hooghe M., Marien S., Oser J. (2017). Great expectations: The effect of democratic ideals on political trust in European democracies. Contemp. Politics.

[74] Fischer J.A.V., Torgler B. (2013). Do positional concers destroy social capital: Evidence from 26 countries. Econ. Inq..

[75] Meng T., Chen H. (2014). A multilevel analysis of social capital and self-rated health: Evidence from China. Health Place.

[76] Xu Z., Sun B. (2020). Influential mechanism of farmers’ sense of relative deprivation in the sustainable development of rural tourism. J. Sustain. Tour..

[77] McNicholl D., McRobie A., Cruickshank H. (2017). Characteristics of Stakeholder Networks Supporting Local Government Performance Improvements in Rural Water Supply: Cases from Ghana, Malawi, and Bolivia. Water Altern. -Interdiscip. J. Water Politics Dev..

[78] Cao W., Zhou S., Wu S., Song C. (2020). Factors influencing farmers’ intentions for urban-rural harmony in metropolitan fringes and regional differences therein. Pap. Reg. Sci..

[79] Xu D., Yong Z., Deng X., Zhuang L., Qing C. (2020). Rural-Urban Migration and its Effect on Land Transfer in Rural China. Land.

